# Supplementary material for: Gender Based Violence against Women in Sub-Saharan Africa: A Systematic Review and Meta-Analysis of Cross-Sectional Studies
Source: Int J Environ Res Public Health. 2020 Feb 1;17(3):903. doi: 10.3390/ijerph17030903 (PMC7037605; doi:10.3390/ijerph17030903)
Supplement: Supplementary file 1 [file ijerph-17-00903-s001.pdf]

International Journal of
*Environmental Research and Public Health*

MDPI

## Supplementary Materials

The following are the seven supplementary figures. Note that the diamond represents the overall combined pooled proportion and the square represents the original studies proportion

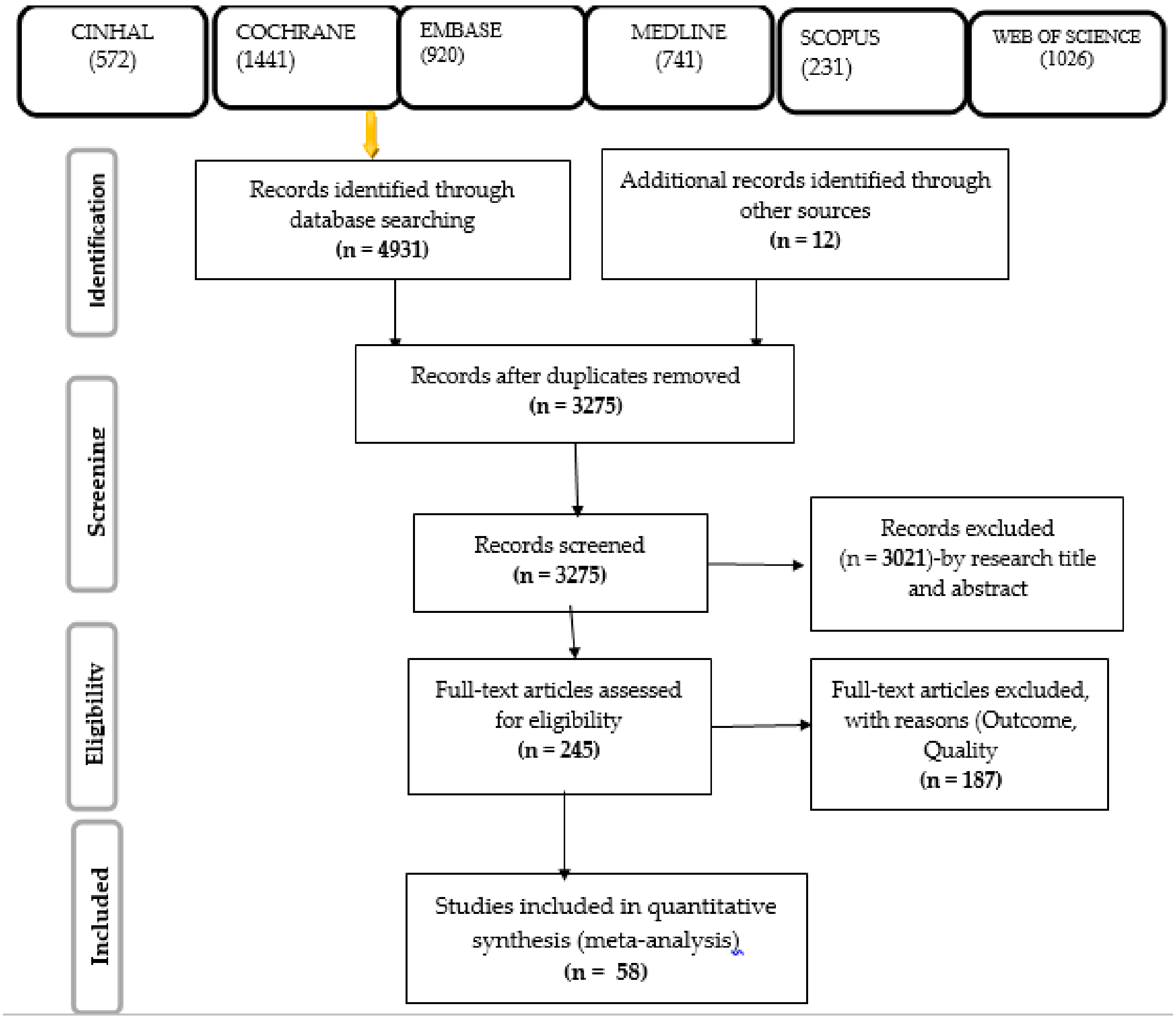


Figure S1: PRISMA flow chart for selection of studies on prevalence of GBV

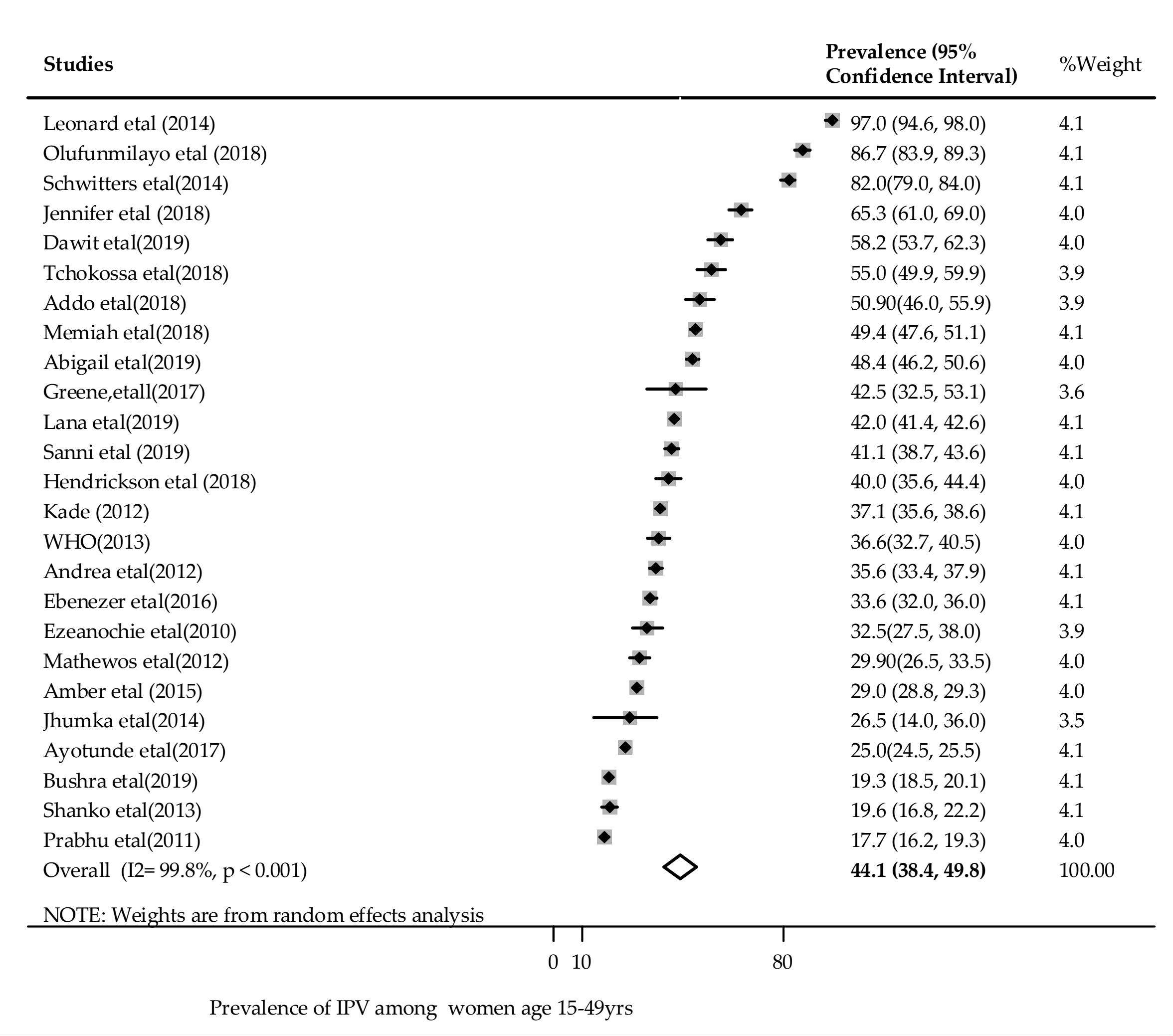


Figure S2. Overall pooled prevalence of IPV among women age 15-49 years old in SSA.

| **Countries** | **Prevalence (95% Confidence interval)** | **%Weight** |
|---|---|---|
| Angola 2015-16 | 33.9 (33.1, 34.7) | 3.5 |
| Benin 2107-18 | 22.4(21.2, 23.7) | 3.5 |
| Burkina Faso 2010 | 11.5 (10.9, 12.1) | 3.5 |
| Burundi 2016-17 | 46.7 (45.5, 47.9) | 3.5 |
| Cameroon 2011 | 51.1 (49.7, 52.5) | 3.5 |
| Chad 2011-12 | 28.6 (27.0, 30.2) | 3.5 |
| Comoros 2012 | 6.4(5.6, 7.3) | 3.5 |
| Congo DR 2013-14 | 50.7 (49.8, 51.6) | 3.5 |
| Cote d'Ivoire 2011-12 | 25.9 (24.8, 26.9) | 3.5 |
| Dominican Republic 2013 | 20.4 (19.5, 21.4) | 3.5 |
| Ethiopia 2016 | 26.3 (25.2, 27.4) | 3.5 |
| Gabon 2012 | 48.6 (47.3, 49.9) | 3.5 |
| Gambia 2013 | 20.0 (18.9, 21.3) | 3.5 |
| Ghana 2008 | 24.4 (21.9, 27.0) | 3.4 |
| Kenya 2014 | 39.4 (38.1, 40.7) | 3.5 |
| Malawi 2015-16 | 33.8 (32.6, 34.9) | 3.5 |
| Mali 2018 | 38.5 (36.9, 40.1) | 3.5 |
| Namibia 2013 | 25.0 (23.3, 26.9) | 3.4 |
| Nigeria 2018 | 21.4 (20.6, 22.9) | 3.5 |
| Rwanda 2014-15 | 34.4 (32.4, 36.4) | 3.4 |
| Sao Tome and Principe 2008-09 | 27.9 (26.1, 29.8) | 3.4 |
| Senegal 2017 | 20.4 (18.8, 22.1) | 3.5 |
| Sierra Leone 2013 | 45.3 (43.2, 47.4) | 3.4 |
| South Africa 2016 | 15.8 (14.9, 16.8) | 3.5 |
| Tanzania 2015-16 | 41.7 (39.0, 44.4) | 3.4 |
| Togo 2103-14 | 22.1 (20.9, 23.3) | 3.5 |
| Uganda 2016 | 46.6 (45.1, 48.1) | 3.5 |
| Zambia 2013-14 | 42.7 (41.4, 44.0) | 3.5 |
| Zimbabwe 2015 | 35.4 (34.2, 36.7) | 3.4 |
| **Overall (I2 = 99.8%, p< 0.001)** | **31.3 (26.3, 36.3)** | 100.0 |

NOTE: Weights are from random effects analysis

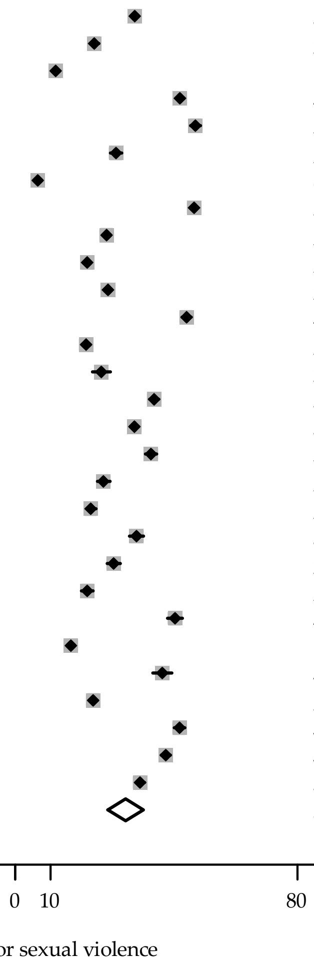


Figure S3: Pooled prevalence of physical or sexual IPV among ever-married women age 15-49 years of old in SSA using DHS surveys

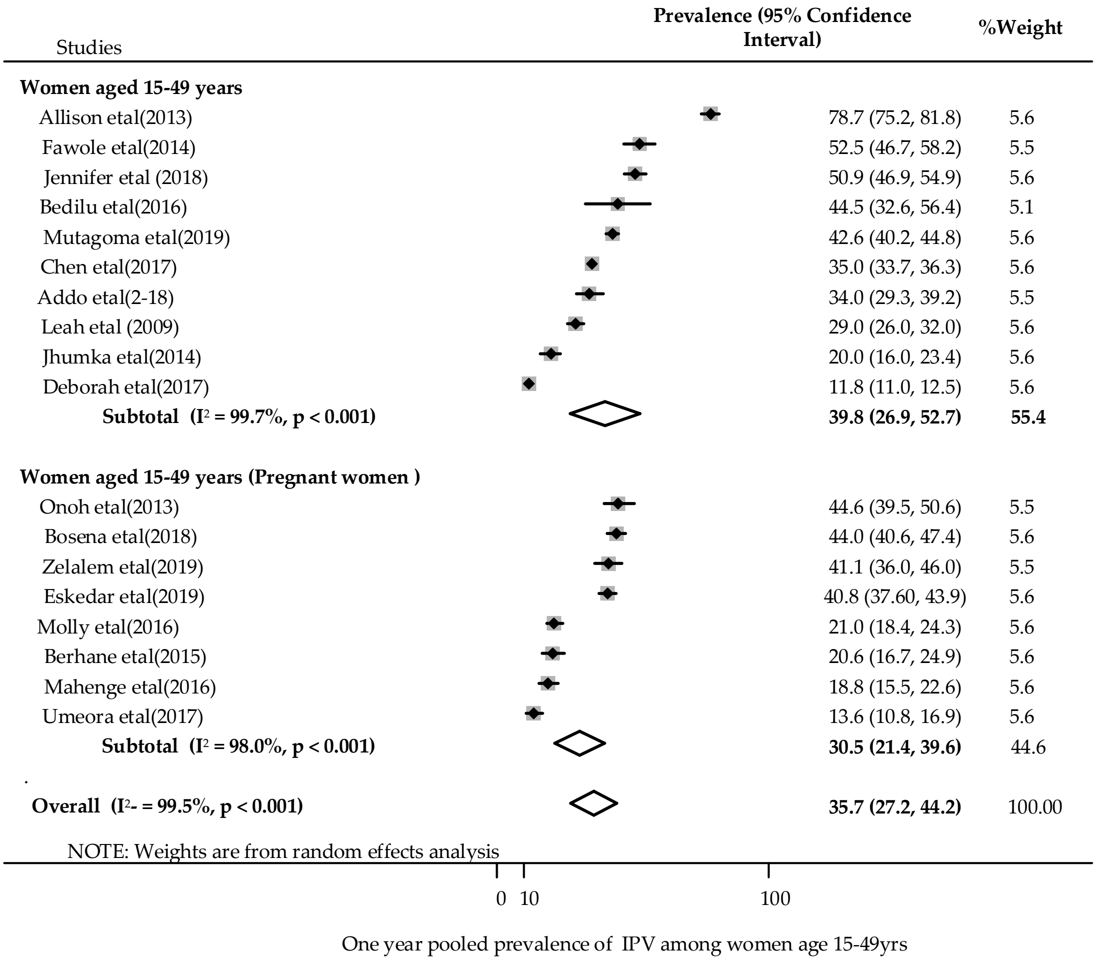


Figure S4: Past one year pooled prevalence of IPV among women age 15-49 years old in SSA

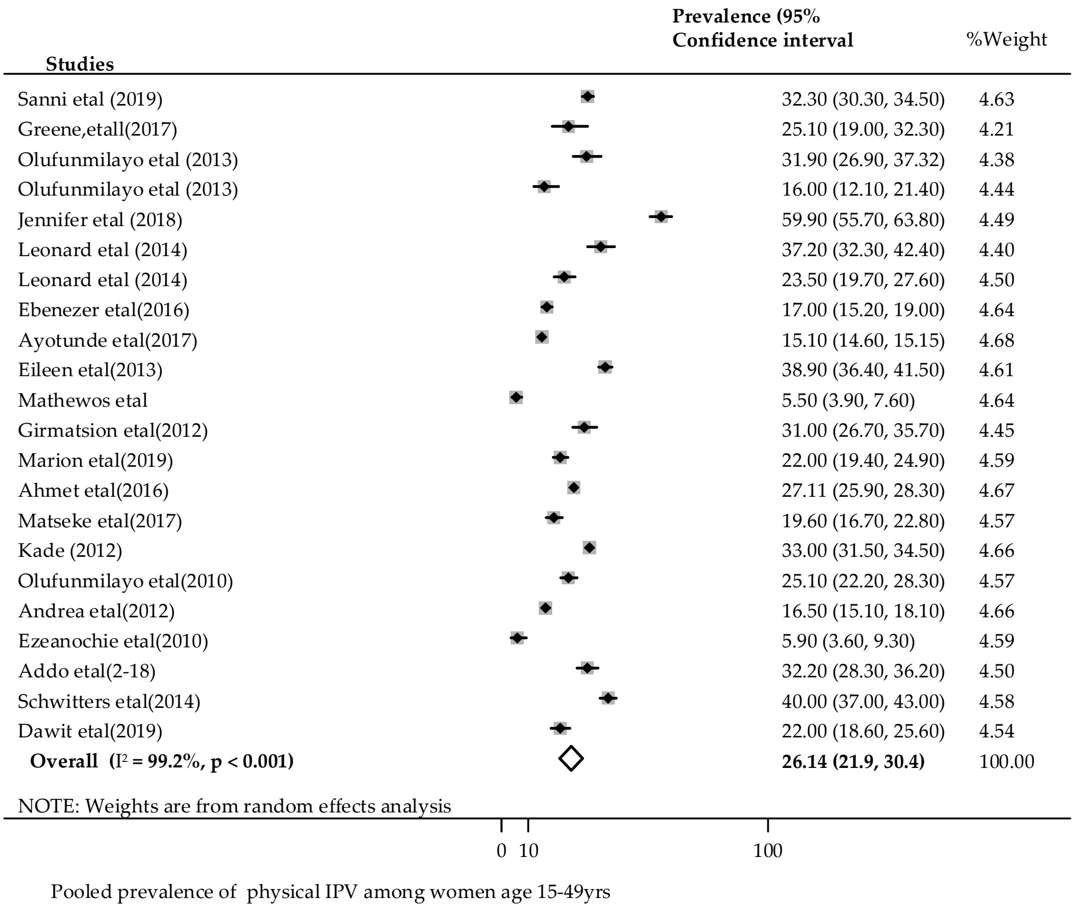


Figure S5: Pooled prevalence of physical IPV among women age 15-49 years old in SSA

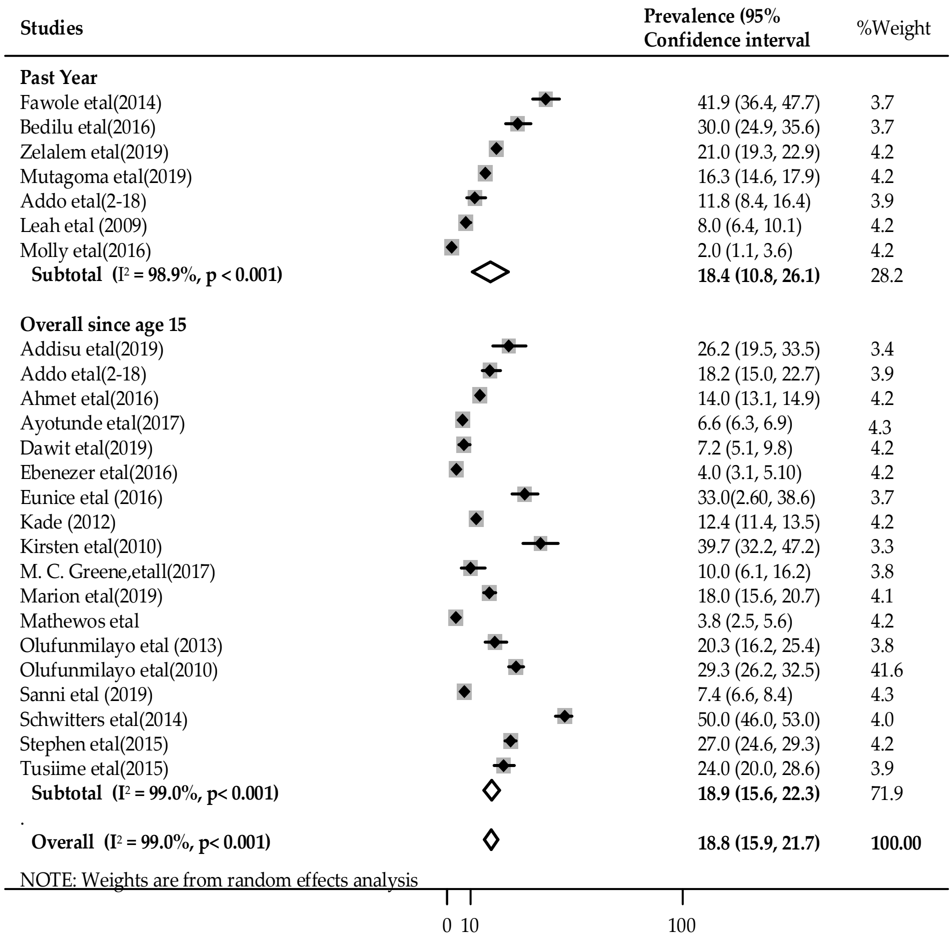


Pooled ssexual IPV Prevalence among women age 15-49yrs

Figure S6: Pooled prevalence of sexual IPV by duration among women age 15-49 years old in SSA

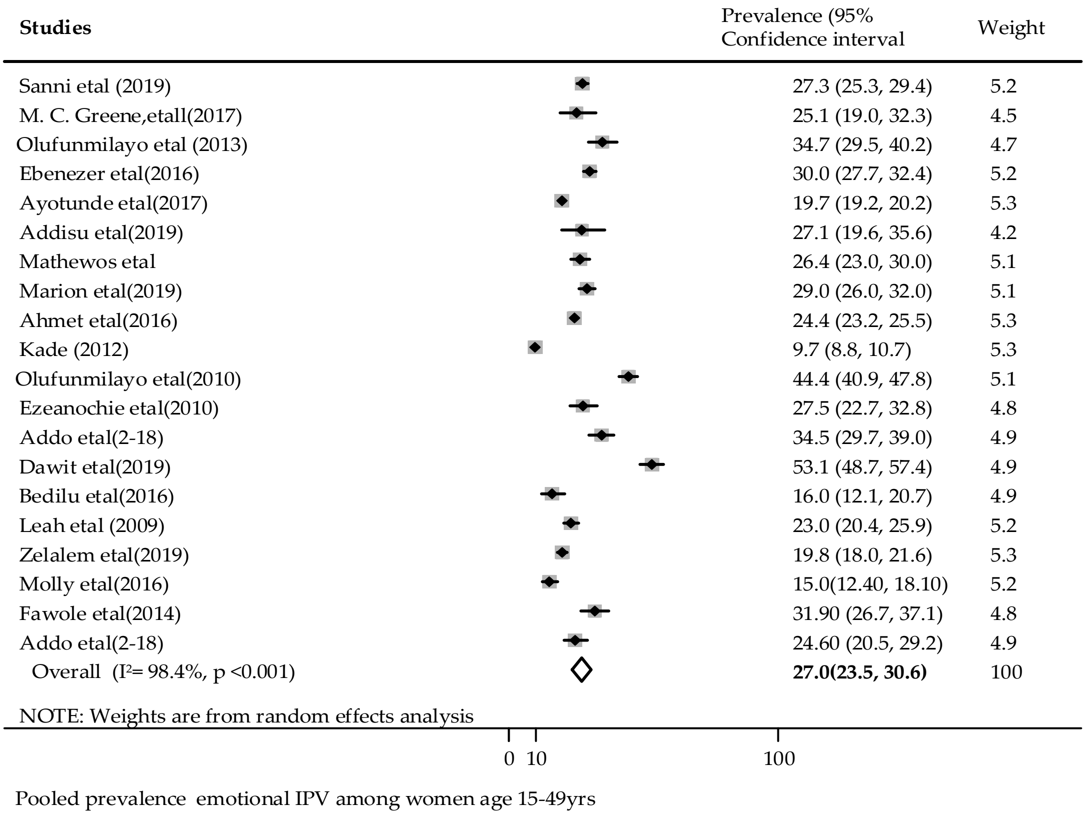


Figure S7: Pooled prevalence of emotional IPV among women age 15-49 years old in SSA

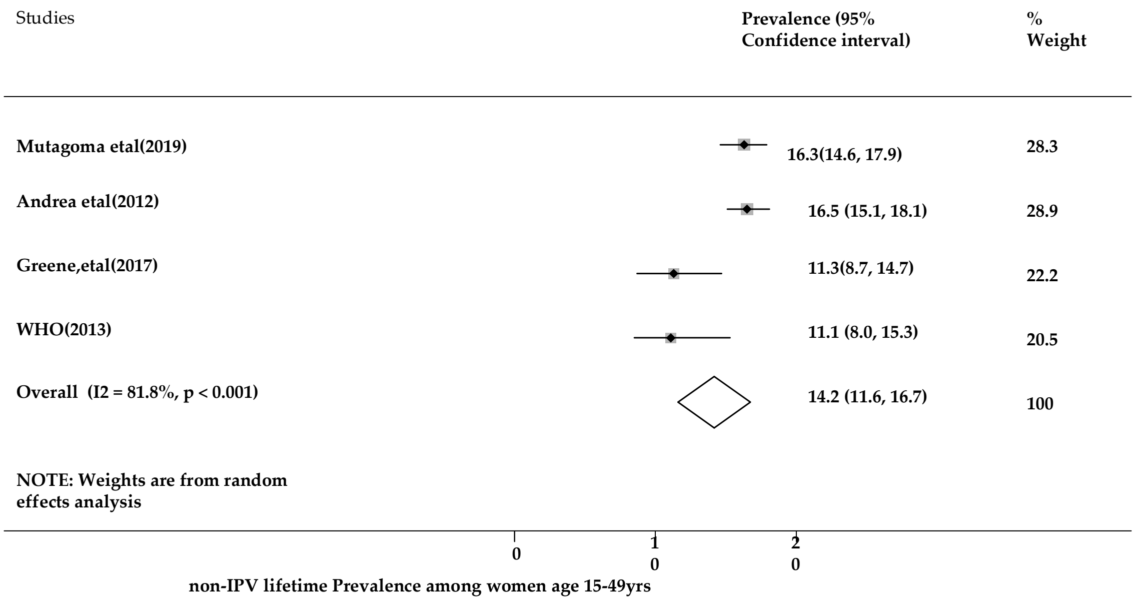


Figure S8: Pooled prevalence of non-IPV among women age 15-49 years old in SSA

**Table S1.** Characteristics of included studies (intimate partner violence (IPV) and non-IPV).

| Authors | Country | Population | Sample Size | Study Design | Outcomes of the Results | Forms of GBV |
|---|---|---|---|---|---|---|
| Bleck et al. (2015) [25] | Selected SSA | Women aged 15–49 years | 44,487 | Cross-sectional | Approximately 29.0% (95% CI 28.8, 29.3) of women reported any physical or sexual IPV in their lifetime | IPV |
| Yaya et al. (2019) [26] | Angola | Women aged 15–49 years | 7669 | Cross-sectional | Overall, more than two-fifths of the women reported experiencing any IPV 41.1% (95% CI 38.7, 43.6): physical IPV 32.3% (95% CI 30.3, 34.5)) was most prevalent, followed by emotional 27.3% (95% CI 25.3, 29.4) and sexual IPV 7.4% (95% CI 6.6, 8.4) | IPV |
| Greene et al. (2017) [27] | 14 countries in SSA | Women aged 15–49 years | 86,024 | Cross-sectional | Any form of lifetime IPV 42.5% (95% CI 32.5, 53.1), IPV was the most prevalent 36.5% (95% CI 26.5, 47.7); non-partner family violence 11.3% (95% 8.7,14.7) and non-family violence 3.2% (95% CI 2.3, 4.3); psychological IPV 25.1% (95% CI 19, 32.3) moderate physical violence 25.6% (95% CI 17.4, 36), severe physical IPV 8.9% (95% CI 5.8, 13.4), any sexual IPV 10% (95% CI 6.1,16.2). | IPV and non-IPV |
| Fawole et al. (2013) [28] | Nigeria | Women (street beggars and traders) | 323 | Comparative cross-sectional study | The lifetime experience of violence against women (VAW) was 66.3% (95% CI 62.5, 70.1) among the beggars and 54.8% (95% CI 52.2, 57.6) among the homemakers ($p < 0.05$). Psychological violence was experienced by 34.7% and 20.8% ($p < 0.05$); physical violence by 31.9% and 16.7% ($p < 0.05$) and sexual by 20.3% and 0.8% ($p < 0.01$) of the beggars and homemakers respectively. | IPV |
| Mootz et al. (2018) [29] | Uganda | Women aged 13 to 49 | 605 | Cross-sectional | Both lifetime and previous year's history: prevalence of experiencing IPV was psychological: 65.3% (95% CI 61%, 69) (life time) and 50.9% (95% 46.9, 54.9) (past 12 months); and physical: 59.9% (95% CI 55.7, 63-8) (lifetime) and 43.8% (95% CI 39.5, 47.8) (one year). | IPV |
| Vinck et al. (2014) [30] | Cote divore | Women aged 15 to 49 years | 950 | Cross-sectional | History of IPV 26.5% (95% CI 14, 36) reported experiencing IPV and 23.4% (95 %CI 16, 41) women reported past-year IPV. | IPV |
| Kirstenet al. (2010) [31] | DR Congo | Women (18–49) | 998 | Cross-sectional | Rates of reported sexual violence were 39.7% (95% CI 32.2, 47.2) | IPV |
| Ajah et al. (2014) [37] | Nigeria | Women aged 15 to 49 years | 836 | Cross-sectional study | The prevalence of domestic violence among rural women was significantly higher than that amongst urban women 97% (95% CI 94.6, 98) versus 81% (95 % CI 77, 84), ($p < 0.001$). In | IPV |

| | | | | | | |
|---|---|---|---|---|---|---|
| | | | | | particular, the prevalence of physical violence was significantly higher among rural women than among urban women 37.2%, (95% CI 32.3, 42.4) versus 23.5 % (95 % CI 19.7, 27.6); (*p* < 0.05). | |
| Adjah et al. (2016) [38] | Ghana | Women aged 15 to 49 years | 1524 | Cross-sectional | The proportion who ever had experienced domestic violence: 33.6% (95% CI 32, 36) emotional 30% (95% CI 27.7, 32.4); physical violence; 17% (95% CI 15.2, 19) and 4% (95% CI 3.1, 5.1) sexual violence | IPV |
| Admasu et al. (2016) [39] | Ethiopia | Women aged 15–49 years | 300 | Cross-sectional | IPV during recent pregnancy was 44.5% (95 % CI, 32.6, 56.4). About 55.5% (95% CI 157, 55.5) of women experienced all the three forms of intimate partner violence during recent pregnancy. Physical 29% (95 % CI, 24, 34.5), sexual 30% (95 % CI, 24.9, 35.6), and psychological 16% (95 % CI, 12, 20.7) | IPV |
| Shanko et al. (2013) [40] | Ethiopian | Women aged 15–49 years | 858 | Cross-sectional | Any experience of violence by an intimate partner was reported by 19.6% (95% CI 16.79, 22.2) and 70.3% of the perpetrators were husbands. | IPV |
| Fawole et al. (2018) [89] | Nigeria | Youth-students | 640 | Cross-sectional | At least one form of GBV was experienced: 86.7% (95% CI 83.9, 89.3) (89.1% of public and 84.8% private schools students (*p* = 0.32)). Psychological violence was the common type of GBV experienced (public—72.5% vs. private—69.2%; *p* = 0.37), while sexual violence was least (public—41.4% vs. private—37.4%; *p* = 0.3) prevalent. | IPV |
| Okenwa et al. (2009) [41] | Nigeria | Women aged 15–49 years | 934 | Cross-sectional | One-year prevalence of IPV was 29% (95% CI 26, 32), with significant proportions reporting psychological 23% (95% CI 20.4, 25.9), physical 9% (95% CI 7.3, 11), and sexual 8% (95% CI 6.4, 10.1) abuse. | IPV |
| Berhane et al. (2015) [90] | Ethiopia | Pregnant women | 422 | Cross-sectional | The prevalence of intimate partner physical violence in pregnancy was 20.6% (95% CI 16.70, 24.90). | IPV |
| Gust et al. (2017) [43] | Kenya | Women age greater than 18-49 years | 7,421 | Cross-sectional | Found that 11.8% (95% CI 11, 12.5) reported physical violence by a sexual partner in the last 12 months. | IPV |
| Kimani et al. (2016) [44] | Kenya | Women aged 15–19 years | 301 | Cross-sectional | Among the respondents, 33% (95% CI 27.6, 38.6) were victims of sexual violence. | IPV |
| Titilayo et al. (2017) [45] | Nigeria | Women aged 15–19 years | 26,997 | Cross-sectional | One-quarter (25%) (95% CI 24.5, 25.5) of the ever married women reported ever experiencing one form of domestic violence or the other (sexual 6.6% (95% CI 6.3, 6.9), physical 15.1% (95% CI 14.6, 15.5) and | IPV |

| | | | | | | |
|---|---|---|---|---|---|---|
| | | | | | psychological/emotional 19.7% (95% CI 19.2, 20.2) | |
| Pitipitan et al. 2013) [46] | South Africa | Women | 1388 | Cross-sectional | A total of 38.9% (95% CI 36.4, 41.5) reported a lifetime history of violence (i.e., ever being hit by a sexual partner). For recent violence, a total of 1140 (82.1%) reported not having been hit and a total of 17.9% (95% CI 15.9, 20.1) women did report having been hit by a sexual partner in the last four months. | IPV |
| Fute et al. (2015) [47] | Ethiopia | Nurses | 660 | Cross-sectional | Prevalence of workplace violence was 29.9% (95% CI 26.5, 33.5) of which physical violence accounted for 5.5% (95% CI 3.9, 7.6), verbal abuse for 26.4% (95% CI 23, 30) and sexual harassment for 3.8% (95% CI 2.5, 5.6). | IPV |
| Fesehan et al. (2012) [48] | Ethiopia | Women | 422 | Cross-sectional | The prevalence of physical violence in the last 12 months and lifetime was 25.5% (95% CI 21.3, 29.8) and 31.0% (95% CI 26.7, 35.7) respectively. The most common forms of physical violence reported included slapping 101 (61.6%) and throwing objects 32 (19.5%). | IPV |
| Fiorentino et al. (2019) [49] | Cameroon | Women ( HIV positive women) | 894 | Cross-sectional | The prevalence of IPV was 29% (95% CI 26, 32) (emotional), 22% (95% CI 19.4, 24.9) (physical), 13% (extreme physical) and 18% (95% CI 15.6, 20.7) (sexual). | IPV |
| Bui et al. (2016) [91] | Zimbabwe | | 5280 | Cross-sectional | Reporting physical violence: 27.11% (95% CI 25.9, 28), sexual 14% (95% CI 13.1, 14.97) and emotional 24.35% (95% CI 23.2, 25.5) | IPV |
| Pack et al. (2013) [92] | Kenya | Sex worker | 619 | Cross-sectional | About 78.7 % (95% CI 75.2, 81.8) of women reporting any IPV in the last 30 days. | IPV |
| Matsekeet al. (2017) [51] | South Africa | HIV positive women | 673 | Cross-sectional | Overall, 56.3% (95% CI 1 52.5, 60) reported having experienced either psychological or physical IPV, and 19.6% (95% CI 16.7, 22.8) reported physical IPV. | IPV |
| Azene et al. (2019) [52] | Ethiopia | Pregnant women | 409 | Cross-sectional | The prevalence of IPV during current pregnancy was found to be 41.1% (95% CI 36, 46). Of this, the prevalence of psychological, physical, and sexual violence was 29.1% (95% CI 27.1, 31.1), 21 % (95% CI 19.26, 22.9) and 19.8% (95% CI 18.0, 21.6) respectively. | IPV |
| Deyessa et al. (2009) [53] | Ethiopia | Women aged 15–49 years | 1994 | Cross-sectional | The lifetime prevalence of any form of IPV was 72% (95% CI 70.0, 73.9). | IPV |
| Gashaw et al. (2018) [54] | Ethiopia | Pregnant women | 720 | Cross-sectional | More than three quarters (80.6%) (95% CI 77.6, 80.5) reported to have a lifetime risk of emotional or physical abuse by their partner or someone | IPV |

| | | | | | | |
|---|---|---|---|---|---|---|
| | | | | | important. The proportion of partner violence during the current pregnancy among ever exposed to IPV was 44% (95% CI 40.6, 47.4). | |
| Berhanie [90] | Ethiopia | Pregnant women | 954 | Cross-sectional | About 40.8% (95% CI 37.6, 43.9) had experienced IPV during their pregnancy period. More than two thirds (68.6%) of cases had been exposed to IPV. | IPV |
| Berestein et al. (2016) [93] | South Africa | Pregnant women | 632 | Cross-sectional | Found that 21% (95% CI 18.4, 24.3) of women reported experiencing ≥1 act of IPV in the past 12 months, including emotional 15% (95% CI (12.4, 18.1), physical 15% (95% CI 12.4, 18.1) and sexual violence 2% (95% CI 1.1, 3.6). Of those reporting any IPV ($n$ = 132), 48% reported experiencing two or more types. Emotional and physical violence was most prevalent among women aged 18–24 years, while sexual violence was most commonly reported among women aged 25–29 years | IPV |
| Pengpid et al. (2016) [55] | 22 Countries (7-Africa) | Women aged 15–49 years | 16,979 | Cross-sectional | Cameroon 51.5%, Ivory Coast 30.2%, Madagascar 15.9%, Mauritius 6.7%, Namibia 21.5%, Nigeria 15.1%, South Africa 18.5%, Tunisia 6.4% | IPV |
| Finnoff et al. (2012)[56] | Rwanda | Women aged 15–49 years | 4066 | Cross-sectional | IPV: 37.1% (95% CI 35.6, 38.6), physical 33% (95% CI 31.5, 34.5), emotional 9.7% (95% CI 8.8, 10.7) and sexual 12.4% (95% CI 11.4, 13.5). | IPV |
| Sabri et al. (2019)[57] | Uganda | Women aged 15–49 years | 7933 | Cross-sectional | Recent IPV victimization was reported by 21.2% (95% CI 18.5, 20.1) of women. | IPV |
| Fawole et al. (2010)[58] | Nigeria | Ever married women | 820 | Cross-sectional | Lifetime prevalence of perpetration of physical abuse was 25.1% (95% CI 22.2, 28.3), while psychological violence was 44.4% (95% CI 40.9, 47.8). Two hundred and forty 29.3% (95% CI, 26.2, 32.5) had ever perpetrated sexual violence. | IPV |
| Agrdah et al. (2012) [59] | Uganda | Students | 980 | Cross-sectional | Overall: 10% (95% CI 8.2, 12.0) exposure to actual physical violence over the previous 12 months. | IPV |
| Perrin et al. (2012) [60] | Somalia | Women aged 15–49 years | 2376 | Cross-sectional | Among women, 35.6% (95% CI 33.4, 37.9) reported adult lifetime experiences of physical or sexual IPV and 16.5% (95% CI 15.1, 18.1) reported adult lifetime experience of physical or sexual non-partner violence. | IPV and Non-IPV |
| Chikhungu et al. (2019) [94] | Malawi | Women aged 15–49 years | 24,562 | Cross-sectional | Found that 42% (95% CI 41.4, 42.6) of ever-married women have experienced some form of violence perpetrated by their current or most recent spouse. | IPV |

| | | | | | | |
|---|---|---|---|---|---|---|
| Wandera et al. (2015) [61] | Uganda | Women aged 15–49 years | 1307 | Cross-sectional | More than a quarter (27%) (95% CI 24.6, 29.3) of women who were in a union in Uganda reported sexual IPV. | IPV |
| Hatcher et al. (2019) [62] | South Africa | Women aged 15–49 years | 2006 | Cross-sectional | Currently partnered men, nearly half (48.4%) (95% CI 46.2, 50.6) perpetrated IPV. | IPV |
| Oumeora (2017) [63] | Nigeria | Women aged 15–49 years | 500 | Cross-sectional | Found that 13.6% (95% CI 10.8, 16.9) of the women had experienced domestic violence in the current pregnancy. | IPV |
| Schneider et al. (2010) [86] | South Africa | Women aged 15–49 years | 425 | Cohort | Found that 13.9% (95% CI 10.8, 17.6) reported IPV at baseline, with physical IPV being the most frequently reported (69.5%). | IPV |
| Tchokossa et al. (2018) [95] | Nigeria | Women aged 15–49 years | 400 | Cross-sectional | Findings showed that 55% (95% CI 49.9, 59.9) of the women have experienced at least one form of violence in their relationship but only 28% of the women who experienced IPV reported the act while 63.7% of those who did not report kept silent because they hoped their partner would change. | IPV |
| Stockl et al. (2010) [96] | Tanzania | Women aged 15–49 years | 1503 | Cross-sectional | Overall physical PV was 9.3% (95% CI 8.3, 10.6) during pregnancy. Seven ($n$ = 88) and twelve per cent ($n$ = 147) of ever-partnered, ever-pregnant women in Dares Salaam ($n$ = 1298) and Mbeya ($n$ = 1205), respectively, reported being physically assaulted during pregnancy by their partner. | IPV |
| Mahenge et al. (2016) [65] | Tanzania | Pregnant women | 500 | Cross-sectional | Overall 18.8% (95% CI 15.5, 22.6) experienced some physical and/or sexual violence during pregnancy. Forty-one women (9%) reported having experienced some physical and/or sexual violence at one to nine months postpartum. | IPV |
| Selin et al. (2019) [87] | South Africa | Adolescent girls and young women | 2533 | Cohort | The prevalence of IPV was nearly one quarter (19.5%) (95% CI 18.0, 21.2) of adolescent girls and young girls (AGYW) experienced any IPV ever (physical or sexual) by a partner. The prevalence of any IPV ever among AGYW aged 13 years to 14 years, 15 years to 16 years, and 17 years to 20 years was 10.8%, 17.7%, and 32.1%, respectively. | IPV |
| Ezeanochie, et al. (2010) [66] | Nigeria | HIV-seropositive pregnant women | 305 | Cross-sectional | The prevalence of IPV among the women was 32.5% (95% CI 27.5, 38.0), with psychological violence being the most common form of violence reported 27.5% (95% CI 22.7, 32.8) and physical violence the least reported 5.9% (95% CI 3.6, 9.3). | IPV |

| | | | | | | |
|---|---|---|---|---|---|---|
| Prabhu, et al. (2011) [97] | Tanzania | women attending VCT | 2436 | Cross-sectional | Overall 17.7% (95% CI 16.2, 19.3) reported IPV during their lifetime. | IPV |
| Fawole et al. (2014) [71] | Nigeria | Female sex worker | 305 | Cross-sectional | The prevalence of VAW preceding the survey was 52.5% (95% CI 46.7, 58.2). Sexual violence was the most common type (41.9 %) ( 95% CI 36.4, 47.7)) of violence experienced, followed by physical violence (35.7%) (95% CI 30.4, 41.3) and psychological (31.9%) (95% CI 26.7, 37.1). | IPV |
| Addo et al.(2017) [98] | Ghana | Women aged 15–49 years | 2000 | Cross-sectional | About 34% (95% CI 29.3, 39.2) of respondents had experienced IPV in the past year, with 11.8% (95% CI 8.4, 16.4), 15.5% (95% CI 12.7, 18.7), and 24.6% (95% CI 0.5, 29.2) reported sexual, physical and emotional respectively. Past year experience of emotional and economic IPV were 24.6% and 7.4% respectively. Where lifetime experience was 50.9% (95% CI 46.0, 55.9), physical 32.2% (95% CI 28.3, 36.2), sexual 18.2 (95% CI 15.3, 22.7) and emotional IPV 34.5% (95% CI 29.7, 39.0). | IPV |
| Chen et al. (2017) [72] | Tanzania | Women aged 15–49 years | 5371 | Cross-sectional | In the past 12 months, there was 35% (95% CI 33.7, 36.3) that reported victimization among the study respondents. | IPV |
| Memiah et al. (2018) [99] | Kenya | Women aged 15–49 years | 3028 | Cross-sectional | Lifetime prevalence was 49.4% (95% CI 47.6, 51.1), ($p < 0.001$). | IPV |
| Schwitter et al. (2014) [73] | Uganda | Female sex workers | 1467 | Cross-sectional | Found that 82% (95 % CI 79, 84) experienced client-initiated GBV and 49% (95 % CI 47, 53) had been raped at least once in their lifetime. Physical violence 40% (95% CI 37, 43), verbal 45% (95% CI 42, 49), and sexual 50% (95% CI 46, 53). | IPV |
| Tusiime, et al. (2015) [74] | Uganda | Young pregnant women | 416 | Cross-sectional | Prevalence of sexual coercion was 24% (95 % 20.0, 28.6) and was higher among those who had non-consensual sexual first time experiences (29.0%) compared with those who had consensual sexual first time experiences (22.6%). | IPV |
| Onoh, et al. (2013) [75] | Nigeria | Pregnant women | 321 | Cross-sectional | Found that, 44.6% (95% CI 39.5, 50.6) reported having been abused in pregnancy. | IPV |
| Falb, et al. (2014) [88] | Côte d'Ivoir | Women aged 15–49 years | 981 | Cohort | Half (49.8%) (95% CI 46.6, 53.1) of all women reported lifetime physical or sexual IPV, and nearly 1 in 5 (18.6%) reported experiencing reproductive coercion. | IPV |

| | | | | | | |
|---|---|---|---|---|---|---|
| Mutagom et al. (2019) [80] | Rwanda | Female sex workers | 1978 | Cross-sectional | A high proportion of female sex workers (FSW's) were physically abused multiple times 42.6% (95% CI 40.2, 44.8). During sex work, 35.6% faced physical violence, and 14.8% faced this physical violence many times. Physical violence happened in the last month preceding the survey in 25.4% FSW's; it occurred in the last 12 months in 49.7% FSWs. When asked about the last time FSWs faced physical violence, most (63.1%) of the perpetrators were clients; however, in 12.5% of (95% CI 11, 14) cases, the perpetrator was a member of law enforcement. A large proportion 18.3% (95% CI 14.6, 17.9) had been sexually abused outside of the family circle. | IPV and non-IPV |
| Bamiwoy et al. (2014) [81] | Multicounty | Women aged 15–49 years | 38,426 | Cross-sectional | The overall prevalence of any form of violence (physical, sexual or emotional) ranged from 30.5% in Nigeria to 43.4% in Zimbabwe; 45.3% in Kenya; 45.5% in Mozambique; 53.9% in Zambia and 57.6% in Cameroon | IPV |
| Yenealem et al. (2019) [100] | Ethiopia | Healthcare workers | 531 | Cross-sectional | The prevalence of workplace violence was found to be 58.2% (95% CI, 53.7, 62.3) in which verbal abuse 53.1% (95% CI 48.7, 57.4) followed by physical attacks 22.0% (95% CI 18.6, 25.6) and 7.2% (95% CI 5.1, 9.8) sexual harassment. Females are most exposed in all forms of workplace violence: verbal abuse 161 (57.1%), physical attack 69 (59.0%) and sexual harassment 38 (100%) when compared with men. | IPV |
| Hendricks et al. (2018) [85] | Tanzania | Female sex workers | 496 | Cross-sectional | Overall 40% (95% CI 35.6, 44.4) of participants experienced recent physical or sexual violence, and 30% recently experienced severe physical or sexual violence. | IPV |
| Garcia- et al. (2013) [4] | Global (56 countries) | Women aged 15–49 years | 11,594 | Cross-sectional | Lifetime prevalence of physical and/or sexual IPV among ever-partnered women by WHO region (African region)—36.6(95% CI 32.7, 40.5); lifetime prevalence of non-partner sexual violence by WHO region—8.5% (95% CI 15.3%, 45.6) proportion of women reporting IPV and/or non-partner sexual violence. | IPV and non-IPV |

**Table S2. Appraisal of Quantitative Studies**

| **Authors** | **Question 1** | **Question 2** | **Question 3** | **Question 4** | **Question 5** | **Question 6** | **Question 7** | **Q 8** |
|---|---|---|---|---|---|---|---|---|
| Bleck et al. (2015) | Yes | Yes | Yes | Yes | Yes | Yes | Yes | Y |
| Yaya et al. (2019) | Yes | Yes | Yes | Yes | Yes | Yes | Yes | Y |
| Greene, et al. (2017) | Yes | Yes | Yes | Yes | Yes | Yes | Yes | Y |
| Fawole et al. (2013) | Yes | Yes | Yes | NO | Yes | Yes | Yes | Y |
| Mootz et al. (2018) | Yes | Yes | Yes | Yes | Yes | Unclear | Yes | |
| Vinck et al. (2014) | Yes | Yes | Yes | Yes | Yes | NO | Yes | N |
| Kirstenet al. (2010) | Yes | Yes | NO | Yes | Yes | Yes | Yes | Y |
| Ajah et al. (2014) | Yes | NO | Yes | NO | Yes | Yes | Yes | N |
| Adjah et al. (2016) | Yes | NO | Yes | Yes | Yes | Yes | Yes | Y |
| Admasu et al. (2016) | Yes | Unclear | Yes | NO | Yes | Yes | Yes | Y |
| Shanko (2013) | Yes | NO | Yes | Yes | Yes | Yes | Yes | Y |
| Fawole et al. (2018) | Yes | Unclear | Yes | NO | NO | Yes | Yes | N |
| Okenwa et al. (2009) | Yes | Yes | Yes | Yes | Yes | Yes | Yes | Y |
| Berhane et al. (2015) | Yes | Yes | Yes | Yes | Unclear | Yes | Yes | Y |
| Gust et al. (2017) | Yes | Unclear | Yes | Yes | Yes | Yes | Yes | Y |
| Kimani et al. (2016) | Yes | Yes | Yes | NO | Yes | Yes | Yes | Y |
| Titilayo et al. (2017) | Yes | Unclear | Yes | Yes | Unclear | Yes | Yes | Y |
| Pitipitan et al. 2013) | Yes | Yes | Yes | Yes | Yes | Yes | Yes | Y |
| Fute et al. (2015) | Yes | Unclear | Yes | Yes | Unclear | Yes | Yes | Y |
| Fesehan et al. (2012) | Yes | Unclear | Yes | Yes | Yes | Yes | Yes | Y |
| Fiorentino et al. (2019) | Yes | No | Yes | Yes | NO | Yes | Yes | Y |
| Bui et al. (2016) | Yes | No | Unclear | Yes | Yes | Yes | Yes | Y |
| Pack et al. (2013) | Yes | No | Unclear | Yes | NO | Yes | Yes | N |
| Matsekeet al. ( 2017) | Yes | No | Unclear | Yes | Yes | Yes | Yes | Y |
| Azene et al. (2019) | Yes | No | Yes | Unclear | Yes | Yes | Yes | Y |
| Deyessa et al. (2009) | Yes | Unclear | Yes | Yes | Yes | Yes | Yes | Y |
| Gashaw et al. (2018) | Yes | Unclear | A | Yes | Yes | Yes | Yes | Y |
| Berhanie et al.( 2019) | Yes | NO | Yes | Yes | Yes | Yes | Yes | Y |
| Berestein et al. (2016) | Yes | Unclear | Yes | Yes | NO | Yes | Yes | Y |
| Pengpid et al. (2016) | Yes | Unclear | Yes | Yes | NO | Yes | Yes | Y |

| | | | | | | | | |
|---|---|---|---|---|---|---|---|---|
| Finnoff et al. (2012) | Yes | Unclear | Yes | Yes | Yes | Yes | Yes | Y |
| Sabri et al. (2019) | Yes | Yes | Yes | Yes | NO | Yes | Yes | Y |
| Fawole et al. (2010) | Yes | Yes | NO | Yes | Yes | Yes | Yes | Y |
| Agrdah et al. (2012) | Yes | Unclear | Yes | Yes | NO | Yes | Yes | Y |
| Perrin et al. (2012) | Yes | Yes | Yes | Yes | Yes | Yes | Yes | Y |
| Chikhungu et al. (2019) | Yes | Unclear | Yes | Yes | Yes | Yes | Yes | Y |
| Wandera et al. (2015) | Yes | Unclear | Yes | Yes | Yes | Yes | Yes | Y |
| Hatcher et al.(2019) | Yes | Yes | Unclear | Yes | Yes | Yes | Yes | Y |
| Oumeora (2017) | Yes | NO | Yes | NO | Yes | Yes | Yes | Y |
| Schneider et al. r(2010) | Yes | NO | No | Yes | Yes | Yes | Yes | Y |
| Tchokossa et al. (2018) | Yes | Yes | Yes | NO | Yes | Yes | Yes | Y |
| Stockl et al. (2010) | Yes | Yes | NO | Yes | NO | Yes | Yes | N |
| Mahenge et al. (2016) | Yes | Unclear | Yes | Yes | NO | Yes | Yes | Y |
| Selin et al. (2019) | Yes | Yes | Yes | Yes | Yes | Yes | Yes | Y |
| Ezeanochie, et al. (2010) | Yes | NO | Yes | NO | Yes | Yes | Yes | N |
| Prabhu, et al. (2011) | Yes | NO | Yes | Yes | Yes | Yes | Yes | Y |
| Fawole et al. (2014) | Yes | Yes | Yes | NO | Yes | Yes | Yes | Y |
| Addo et al(2017) | Yes | Unclear | Yes | Yes | Yes | Yes | Yes | Y |
| Chen et al. (2017) | Yes | Yes | Yes | Yes | Yes | Yes | Yes | Y |
| Memiah et al. (2018) | Yes | NO | Yes | Yes | Yes | Yes | Yes | |
| Schwitter et al. (2014) | Yes | NO | Yes | Yes | NO | Yes | Yes | N |
| Tusiime, et al. (2015) | Yes | NO | Yes | Yes | NO | Yes | Yes | N |
| Onoh, et al. (2013) | Yes | unclear | Yes | NO | Yes | Yes | Yes | Y |
| Falb, et al. (2014) | Yes | Yes | Yes | Yes | Yes | Yes | Yes | Y |
| Mutagom et al. (2019) | Yes | Yes | Yes | Yes | Yes | Yes | Yes | Y |
| Bamiwoy et al. (2014) | Yes | Yes | Yes | Yes | Yes | Yes | Yes | Y |
| Yenealem et al. (2019) | Yes | Yes | Yes | Yes | Yes | Yes | Yes | Y |
| Hendricks et al. (2018) | Yes | Yes | Yes | Yes | Yes | Yes | Yes | Y |
| Garcia- et al. (2013) | Yes | Yes | Yes | Yes | Yes | Yes | Yes | Y |

**CASP Key Questions**

1. Did the study address a clearly focused issue?
2. Was the participants of the study recruited in an acceptable way?
3. Was the outcome accurately measured to minimise bias?
4. Was the sampling appropriate for the study
5. What are the results of the study?
6. How precise the tools used to measure the results?
7. Do you believe the results?
8. Can the results be applied to the local population?
9. Do the results of the study fit with other available evidence?
10. What are the implications of this study for practice?
